# Comparison of Single Intraoperative Dose of Dexamethasone on Glycemic Profile in Postoperative Diabetic and Non-diabetic Patients

**DOI:** 10.5812/aapm-161467

**Published:** 2025-04-30

**Authors:** Shetty Shyvi Ravindra, Ahlam Abdul Rahman, Rashmi R. Aithal, Sonal Bhat, Akshatha D.

**Affiliations:** 1 Department of Anaesthesiology, Kasturba Medical College Mangalore, Manipal Academy of Higher Education, Manipal, India

**Keywords:** Dexamethasone, Blood Glucose, PONV

## Abstract

**Background:**

This is a prospective observational study. Dexamethasone is commonly associated with postoperative hyperglycemia. There is limited data on the glycemic effect of dexamethasone among the diabetic population.

**Objectives:**

In the current investigation, postoperative glucose levels were measured in both diabetic and non-diabetic individuals, and then a single dose of intraoperative dexamethasone was administered.

**Methods:**

A total of 86 participants, with ASA I/II, were categorized into two groups: Diabetic and non-diabetic. Each group consisted of 43 individuals. The participants' ages ranged from 18 to 70 years. During the operation, a single dosage of dexamethasone, with a maximum of 8 milligrams, was provided intraoperatively. “Postoperative nausea and vomiting” (PONV), random blood glucose (RBG), and pain ratings were recorded before surgery, immediately after surgery, after 12 hours, and 24 hours following surgery. Preoperative blood glucose levels were also recorded. The “Chi-square test and the unpaired *t*-test” were used for comparison and to analyze the data. A significance level of P < 0.05 was deemed significant.

**Results:**

There was a statistically significant variance in RBG levels between diabetics and non-diabetics (P = 0.001). At various time periods, there was no correlation between the severity of PONV and diabetes among the participants. At various time intervals, the length of the procedure and the pain levels were equivalent to one another. In each group, there was a significant rise in RBG up to 12 hours, followed by a decline after 24 hours to a level similar to preoperative values.

**Conclusions:**

A single dose of intraoperative “dexamethasone” was associated with transient hyperglycemia postoperatively up to 12 hours, which was more pronounced among the diabetic population and without major adverse effects like PONV in either group.

## 1. Background

Steroids are used in a wide variety of therapeutic settings owing to their anti-inflammatory and immune-modulating effects, which have led to their widespread utilization. Side effects of using steroids range from acne to diabetes and potentially life-threatening heart diseases if not appropriately treated (1). Dexamethasone is a “glucocorticoid” often given for the management of “asthma, chronic obstructive pulmonary disease, acute respiratory distress syndrome, sepsis, septic shock in the intensive care unit, migraine, depression, multiple sclerosis, thyroiditis, and coronary syndromes,” among others (2). The practice guidelines of the American Society of Anesthesiologists in 2013 concluded that using dexamethasone is advantageous during surgery to reduce “postoperative nausea and vomiting” (PONV) (3). In addition, it reduces postoperative opioid requirement, sleep disturbance, and improves the quality of postoperative recovery (4). Dexamethasone is widely considered to be the best perioperative medication due to its low cost, widespread availability, excellent performance for PONV, appetite-promoting properties, and association with quicker release from day surgery units (5). Dexamethasone has been proven effective at a dose ranging from 4 mg to 10 mg administered intravenously in surgery (6). The intraoperative administration of 8 mg intraperitoneal (IP) dexamethasone in patients undergoing laparoscopic cholecystectomy was associated with a reduction in PONV within the first 24 hours; however, this effect did not reach statistical significance. Notably, IP dexamethasone significantly decreased postoperative pain (POP) and the requirement for analgesics during the same period, demonstrating superior efficacy compared to both the intravenous (IV) dexamethasone and control groups (7). On the other hand, the administration of a single dose of "perioperative dexamethasone" is linked to a rise in infection risk as well as hyperglycemia (8, 9). In addition, those who have poor glycemic control and hyperglycemia are more likely to have complications from wounds and infections at surgical sites (10). Additionally, postoperative infections have been speculated among patients with diabetes (11). Nevertheless, there have been reports of increased blood glucose concentrations among non-diabetics after perioperative administration of dexamethasone (9, 12), with limited data among the diabetic population.

## 2. Objectives

The purpose of this research was to investigate the impact that “a single intraoperative dosage of dexamethasone” had on the glycemic profile of diabetic and non-diabetic individuals after they had undergone surgery.

## 3. Methods

### 3.1. Study Design and Setting

Between the months of December 2022 and December 2024, prospective observational research was carried out at the "Department of Anaesthesiology of a teaching hospital in South India" that provided tertiary care. After receiving consent from the "Institutional Scientific and Ethics Committee", the research was conducted, and prior informed consent was obtained from each patient before they were enrolled as participants in the study.

### 3.2. Sample Size and Study Participants

A pooled standard deviation of 2.4, a power of 95%, an alpha error of 5%, and a confidence interval of 95% were used to generate a minimum sample size of 43 individuals assigned to each group. Under general anesthesia, the sample frame consisted of all adult patients, both diabetic and non-diabetic, who were scheduled to have elective procedures. Participants in the trial were children and adults with “type 2 diabetes who were between the ages of 18 and 70 years old, had an ASA physical status of I or II”, and gave their informed consent. Patients on steroid therapy, those with malignancy, any history of dexamethasone allergy, peptic ulcer, hepatorenal dysfunction, and obstetric patients were excluded from the research.

### 3.3. Methodology

After obtaining informed consent, patients (henceforth participants) were conveniently selected and categorized into two groups: Participants in the study group had type 2 diabetes mellitus, and those not having type 2 diabetes mellitus were grouped as controls. Participants were shifted to the operation theatre (OT) complex 30 minutes before surgery. In the OT complex, “standard monitors (non-invasive blood pressure, electrocardiogram, pulse oximetry, and capnogram) were connected, and an appropriately sized IV cannula was secured for drug and fluid administration”. Their preoperative vitals such as “heart rate, blood pressure, respiratory rate, oxygen saturation, and mean arterial pressure” were recorded using a multi-parameter monitor. In addition, “preoperative random blood glucose levels were recorded. All patients were preloaded with 5 mL/kg of crystalloids (Ringer lactate)”. Participants were administered a loading dose of dexmedetomidine about 15 minutes before induction of anesthesia. This was followed by “intravenous administration of 0.02 mg/kg midazolam, 2 µg/kg fentanyl, 0.2 mg glycopyrrolate.” Patients were preoxygenated with 100% oxygen using an appropriately sized face mask. Induction of anesthesia was then initiated by injection of propofol 2 mg/kg in a titrated dose. Immediately after the induction, a “single dose of intravenous dexamethasone (0.15 mg/kg to a maximum of 8 mg)” was administered. Intubation was facilitated with an injection of rocuronium 0.8 mg/kg. Participants were intubated after 90 seconds with an appropriately sized endotracheal tube, after confirming bilateral equal air entry, and it was ensured that the endotracheal tube was fixed. Participants were maintained with oxygen: Nitrous (50:50) and an appropriate amount of sevoflurane to maintain an MAC of 1. After the completion of the surgery, study participants were shifted to the postoperative recovery room for observation for 24 hours. The parameters recorded were random blood glucose (RBG) levels, PONV, and pain using “Visual Analog Scores (VAS) preoperatively, immediately postoperative, after 12 hours, and after 24 hours respectively. The RBG levels were measured by means of a calibrated glucometer. Pain was assessed using the “VAS with scores ranging from 0 “no pain” to 10 “the worst imaginable pain.” The severity of PONV was assessed using a 4-point verbal descriptive scale which consists of the following scores: Score 0 = no PONV: No complaint of nausea or vomiting; Score 1 = mild PONV: Patient complains of nausea but refuses antiemetic treatment; Score 2 = moderate PONV: Patient complains of nausea and needs antiemetic treatment; and Score 3 = severe PONV: Patient complains of nausea with an episode of vomiting requiring antiemetic treatment”. The primary outcomes included RBG levels at different time intervals with a secondary outcome of severity of PONV and pain scores. The information was analyzed using SPSS for Windows (version 26 of SPSS released by IBM Corporation in Armonk, New York). In order to compare the continuous data across the groups, the unpaired *t*-test was used, and the chi-Square test was utilized in order to compare the categorical data from each group. Graphs and tables were used in the presentation of the data. P < 0.05 was chosen as the criterion of significance for this study.

## 4. Results

The mean age and distribution of gender between the groups were comparable (P > 0.05). More diabetic participants belonged to ASA I (P = 0.01), and the time taken to complete the surgery was comparable between the groups. Random blood glucose levels were found to be significantly elevated among diabetics when compared to non-diabetics at different time intervals (P = 0.001). Furthermore, there was no association between the severity of PONV and the diabetic status of participants between the groups at different time intervals (P > 0.05). Additionally, pain scores were found to be comparable between groups at “different time intervals (P > 0.05)”.

Table 1 compares the demographic characteristics between diabetic and non-diabetic participants. The mean age was slightly higher in the diabetic group, but the difference was not statistically significant (P = 0.051). Gender distribution was similar between groups (P = 0.99). A significant difference was observed in ASA physical status, with all diabetic patients classified as ASA II, while the majority of non-diabetics were ASA I (P = 0.01). The duration of surgery was comparable between the two groups (P = 0.80).

**Table 1.** Comparison of Demographic Characteristics Among Participants Between Groups ^a^

Variables	Diabetic	Non-diabetic	P-Value
**Age**	47.16 ± 10.04	42.6 ± 12.69	0.051 (NS)
**Gender**			
Male	27 (54)	28 (56)	0.99
Female	23 (46)	22 (44)	NS
**ASA**			0.01 ^b^
ASA I	0	29 (58)	
ASA II	50 (100)	21 (42)	
**Duration of surgery**	1.95 ± 0.64	1.98 ± 0.53	0.8

^a^ Values are expressed as mean ± SD or No. (%).

^b^ Statistically significant using chi-square test.

**Figure 1.**
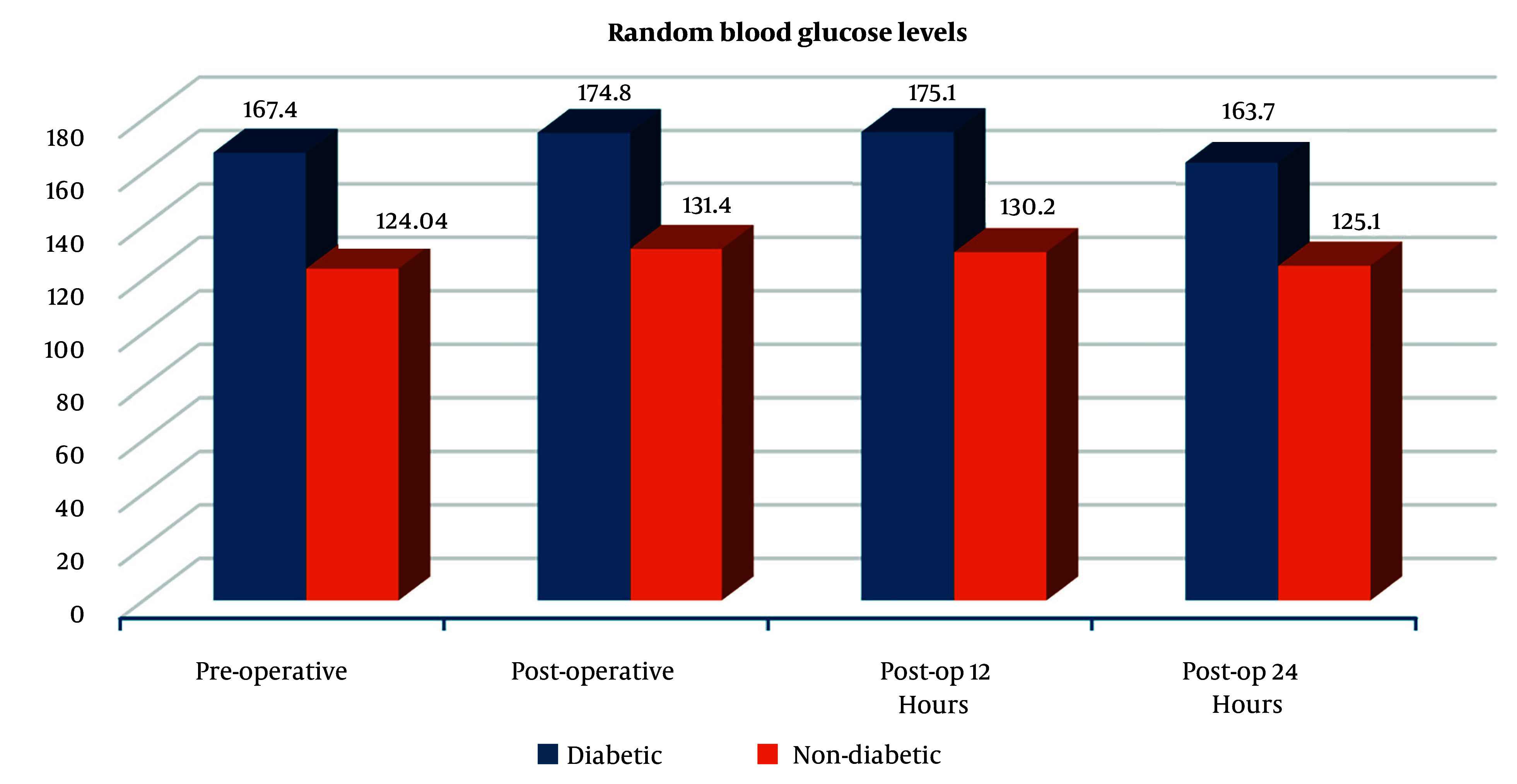
Mean random blood glucose (RBG) levels at different time intervals between groups

Figure 1 illustrates the trend in RBG levels at four time points — pre-operative, post-operative, 12 hours post-op, and 24 hours post-op — among diabetic and non-diabetic patients. Diabetic patients consistently exhibited higher blood glucose levels than non-diabetic patients across all time points. In the diabetic group, glucose levels peaked post-operatively (174.8 mg/dL) and remained elevated at 12 hours (175.1 mg/dL), followed by a slight decline at 24 hours (163.7 mg/dL). In contrast, non-diabetic patients showed relatively stable glucose levels, with a modest post-operative increase (from 124.04 mg/dL pre-op to 131.4 mg/dL), followed by a gradual decrease to 125.1 mg/dL at 24 hours.

Table 2 shows that there is no significant difference in the incidence of PONV between diabetic and non-diabetic participants at any time point (postoperative, 12 hours, and 24 hours). The P-values for all comparisons are greater than 0.05, indicating no statistical significance.

**Table 2.** Distribution of Postoperative Nausea and Vomiting Among Participants Between Groups ^a^

Variables	Diabetic	Non-diabetic	Total	P-Value
**Postoperative**				0.33 (NS)
PONV 0	16 (32)	15 (30)	31	
PONV 1	26 (52)	21 (42)	47	
PONV 2	8 (16)	14 (28)	22	
Total	50 (100)	50 (100)	100	
**Post 12 (h)**				0.86 (NS)
PONV 0	18 (36)	17 (34)	35	
PONV 1	25 (50)	24 (48)	49	
PONV 2	7 (14)	9 (18)	16	
Total	50 (100)	50 (100)	100	
**Post 24 (h)**				0.97 (NS)
PONV 0	20 (40)	19 (38)	39	
PONV 1	24 (48)	25 (50)	49	
PONV 2	6 (12)	6 (12)	12	
Total	50 (100)	50 (100)	100	

Abbreviations: PONV, postoperative nausea and vomiting; NS, not significant using chi-square test.

^a^ Values are expressed as No. (%).

Table 3 compares the mean pain scores between diabetic and non-diabetic participants at three time points: Pre-operative, post-operative, and 12 hours post-operatively. There were no significant differences in pain scores between the two groups at any time point (P > 0.05). These findings suggest that diabetes does not significantly affect pain perception in the postoperative period.

**Table 3.** Mean Pain Scores of Participants Between the Groups ^a^

Variables	Diabetic	Non-diabetic	P-value
**Pre-op**	1.42 ± 0.49	1.38 ± 0.49	0.68 (NS)
**Post-op**	146 ± 0.5	1.4 ± 0.49	0.5 (NS)
**post-op 12 hours**	1.36 ± 0.48	1.28 ± 0.45	0.39 (NS)

Abbreviation: NS, not significant using unpaired *t*-test.

^a^ Values are expressed as mean ± SD.

Table 4 presents a comparison of glucose levels between diabetic and non-diabetic patients across four time points: Pre-operative (pre-op), post-operative (post-op), 12 hours post-op, and 24 hours post-op. Both diabetic and non-diabetic groups exhibited significant post-operative glucose fluctuations, though diabetics showed more variability in their post-op levels. These changes highlight the impact of surgery on glucose metabolism, with notable differences between the two groups.

**Table 4.** Comparison of Mean Random Blood Glucose at Different Time Intervals for Diabetic and Non-diabetic Participants ^a^

Variables	Diabetic	F	P-Value	Non-diabetics	F	P-Value
**Time intervals**		6.41	0.005 ^b^		112	0.001 ^b^
Pre-op	167.4 ± 35.1			124 ± 17.6		
Post-op	174.8 ± 32.3			131.4 ± 16.1		
Post-op 12 (h)	175.1 ± 28.4			130.2 ± 15.5		
Post-op 24 (h)	163.7 ± 23.8			125.1 ± 15.1		
**Pairwise comparison**		-			-	
Pre-op vs. post-op	-		0.001 ^c^	-		0.001 ^c^
Post-op vs. post-op 12 (h)	-		0.99	-		0.99
Post-op 12 (h) vs. post-op 24 (h)	-		0.001 ^c^	-		0.001 ^c^
Post-op 24 (h)vs Pre-op	-		0.97	-		0.99

^a^ Values are expressed as mean ± SD.

^b^ Statistically significant using one-way ANOVA.

^c^ Statistically significant adjust using Bonferronis correction.

## 5. Discussion

The present comparative study was conducted to analyze and compare the glycemic effects of a single intraoperative dose of dexamethasone among diabetics and non-diabetics. Compared to the non-diabetic group, the diabetic group experienced a transient increase in “blood glucose levels immediately following surgery, 12 hours after surgery, and 24 hours after surgery after receiving a single IV dose of "dexamethasone (0.15 mg/kg to a maximum of 8 mg)" right after the induction of anesthesia”. In addition, PONV was found to be comparable between both groups at different time intervals. Though not statistically significant, participants in the diabetic group had comparatively higher pain scores than participants in the non-diabetic group. Furthermore, the highest blood glucose levels were observed in both groups at 12 hours after surgery, followed by reaching similar baseline values 24 hours after surgery.

In the present study, a transient elevation of blood glucose levels was detected among participants in the diabetic group compared to the non-diabetic group. Our findings were similar to Tien et al. (13) and Hans et al. (as cited by Joshi et al.) (14), who reported a comparable surge in blood glucose levels among diabetic patients. Though the findings by Hans et al resonate with our findings, we observed that Hans et al did not have a control group, which raises concern if the diabetic status of a patient had any effect on the glycemic values. In addition, Hans et al. (as cited by Joshi et al.) in their research observed the blood glucose levels only up to 6 hours postoperatively, whereas it is established that blood glucose levels can rise beyond 6 hours (14). On the other hand, Wasfie et al. (15) reported a similar rise in blood glucose levels among participants who were given dexamethasone (among diabetic and non-diabetic), suggesting that type 2 diabetic patients are equally susceptible to the “hyperglycemic effects of dexamethasone as non-diabetics”. In yet another study, Patil et al. (16) and Herbst et al. (17) observed a temporary rise in blood glucose levels in both diabetic and non-diabetic individuals after receiving a single dose of dexamethasone during the perioperative period. On the contrary, Abdelmalak et al. (18) found that blood glucose levels were higher in non-diabetic subjects given dexamethasone compared to type 2 diabetic patients. This difference in results could be attributed to a change in the methodology, viz., different study populations, different timing and frequency of measuring blood glucose levels, tailored to fulfill their objectives which focused on obtaining intra-operative findings. In yet another study, Peter et al found that non-diabetic individuals saw a rise in their postoperative blood glucose levels after the “administration of a single dose of dexamethasonee”. This finding further demonstrates that diabetes does not cause postoperative glycemia.

The follow-up in our study was conducted up to 24 hours, and even at 24 hours after surgery, we observed that blood glucose values were elevated among patients in the diabetic group. In the present study, a maximum blood glucose level was observed at 12 hours after surgery. Hans et al. (as cited by Joshi et al.) (14) in their study monitored blood glucose levels for up to 6 hours, while Wasfie et al. (15) monitored the rise in blood glucose levels for 12 hours. Tien et al. (13) and Herbst et al. (17) continued the observation for up to 24 hours and found that patients on dexamethasone in the diabetic group had elevated blood glucose levels. A “systematic review and meta-analysis by Pang et al. (19) reported peak glucose levels within 24 hours of surgery, while Peter et al. continued the observation for 72 hours and reported the peak levels at 24 hours” post-surgery among non-diabetic participants (20). The findings from the above studies revealed that maximum blood glucose levels were observed either within or at 24 hours after surgery, which was in contrast to our findings except for Wasfie et al. (15), who found the peak levels at 10 hours post-surgery. O'Connell et al. (21) reported peak blood glucose levels to occur between 24 hours to 72 hours after surgery.

In the present study, we observed that PONV among participants in both groups was comparable immediately after surgery, 12 hours, and 24 hours after surgery. Though statistically not significant, it was found that the majority of the participants in both groups had mild PONV. Dexamethasone is known to reduce PONV episodes among participants. A randomized trial (DREAMS trial) conducted among a large sample revealed that a single dose of dexamethasone is potent enough to reduce the incidence of PONV (22). The decrease in PONV may be related to its anti-inflammatory activity, which effectively lowers local inflammatory responses postoperatively, reducing afferent stimulation to the vomiting center and hence decreasing PONV.

In the present study, we attempted to determine pain scores between “groups after a single-dose administration of dexamethasone. We found no significant difference in mean pain scores between diabetic and non-diabetic participants immediately after surgery, 12 hours, and 24 hours after surgery. This was not in line with reports from a systematic review and meta-analysis, which observed that participants receiving dexamethasone had lower pain scores 2 hours post-surgery”. However, we need to consider this comparison with caution since the report included participants undergoing surgeries irrespective of their glycemic status (23).

This study found that preoperative blood glucose levels were similar to those measured 24 hours post-surgery in both “diabetic and non-diabetic participants”. However, prior research indicates that “dexamethasone's effect on blood glucose” typically persists for up to 72 hours. The restoration of blood glucose to preoperative levels may be attributed to a reduced time of operation in both groups, suggesting that the research included patients having modest surgical procedures.

Our study had some limitations. First, blood glucose measurements were limited to only 24 hours postoperatively, whereas there is evidence that the hyperglycemic activity of dexamethasone exceeds up to 72 hours. Second, dexamethasone was administered to participants with and without diabetes. Incorporation of a control arm in each group would have enabled us to determine if blood glucose levels were affected by the diabetic status of a participant. Third, the type of surgery was not controlled since different types of surgeries elicit different stress responses.

The research concludes that, given its limits, a single perioperative dosage of dexamethasone post-induction caused transitory hyperglycemia in diabetic participants compared to non-diabetic individuals. Adverse effects such as PONV were similar across the groups at various time intervals. No notable change was seen in pain levels or surgical duration. Within-group analysis demonstrated an apparent rise in the level of blood glucose from preoperative measurements to 12 hours postoperatively, followed by a considerable reduction after 24 hours postoperatively, returning to levels comparable to preoperative values. The highest peak readings of blood glucose were seen 12 hours after surgery.

### 5.1. Conclusions

It can be concluded that within the limitations of the present study, a single dose of dexamethasone administered preoperatively before the procedure resulted in transient hyperglycemia among participants with diabetes compared to those without diabetes. Adverse effects like PONV were comparable between the groups at different time intervals. There was no significant difference in pain scores and duration of surgery. Within-group analysis revealed a significant increase in blood glucose levels from preoperative to 12 hours postoperatively, followed by a significant decrease after 24 hours postoperatively, which were similar to preoperative levels. In addition, the maximum peak blood glucose levels were found at 12 hours postoperatively.

Transient hyperglycemia in non-diabetics is a temporary rise in blood glucose often triggered by acute illness, stress, or medications. Clinically, it signals increased physiological stress and is linked to worse outcomes in hospitalized patients. It may also unmask prediabetes or signal future diabetes risk. Recognizing and managing it can guide prognosis, prevent complications, and prompt appropriate follow-up after recovery.

## Data Availability

The dataset presented in the study is available on request from the corresponding author during submission or after publication. The data are not publicly available due to privacy.

## Clinical Trial Registration Code:

CTRI/2023/10/058442.
